# Outcomes of Bladder Washout for the Treatment of Recurrent Urinary Tract Infections After Renal Transplantation

**DOI:** 10.7759/cureus.58556

**Published:** 2024-04-18

**Authors:** Spencer Mossack, Ari M Spellman, Serafino A Lagalbo, Carlos A Santos, Vasil Peev, Samuel Saltzberg, Edie Chan, Oyedolamu Olaitan

**Affiliations:** 1 Urology, Rush University Medical Center, Chicago, USA; 2 Urology, Mount Sinai Hospital, New York, USA; 3 Medicine, Rush University Medical Center, Chicago, USA; 4 Infectious Disease, Rush University Medical Center, Chicago, USA; 5 Nephrology, Rush University Medical Center, Chicago, USA; 6 Transplantation, Rush University Medical Center, Chicago, USA; 7 Surgery, Rush University Medical Center, Chicago, USA

**Keywords:** cystoscopy, recurrent urinary tract infections, bladder washout, urinary tract infection, renal transplantation

## Abstract

Background

Current literature suggests that anywhere from 2.9-27% of renal transplant recipients (RTR) will develop recurrent urinary tract infections (UTIs) (≥2 UTIs over six months or ≥3 UTIs over 12 months). Recurrent UTIs are of particular importance to RTR given its increased risk for allograft fibrosis and overall patient survival. Alternative solutions are needed for the management of recurrent UTIs, especially given the vulnerability of RTR to UTIs. We hypothesize that bladder washout (BW) reduces the incidence and recurrence of UTIs in RTR.

Methods

This is a retrospective study evaluating the utility of BW procedures on RTR diagnosed with recurrent UTIs between December 2013 and July 2021 at a single center.

Results

A total of 106 patients were included in the study with a total of 118 BW performed. 69% of patients were successfully treated with BW, meaning they no longer met the criteria for recurrent UTIs (<1 UTI) in the six-month post-BW period. The mean number of UTIs was 2.76 (range 2-7) before the BW and 1.16 (range 0-5) after the BW. On average, there were 1.60 fewer UTIs in the post-BW period compared to the pre-BW period (p<0.0001). There is no statistically significant difference in success rates stratified by bacterial class (p=1) or antimicrobial resistance class (p=0.6937).

Conclusion

BW decreased the incidence of UTIs in the six-month post-operative period as nearly 70% of patients did not have UTI recurrence. This data provides evidence that BW may have utility in transplant recipients with recurrent UTIs. We hope this will stimulate further prospective randomized studies in this area.

## Introduction

Urinary tract infections (UTIs) are the most common bacterial infections affecting renal transplant recipients (RTR). Risk factors for UTIs in RTR include older age, female sex, recipient from a deceased donor, ureteral stents, and diabetes mellitus [1,2]. Pretransplant urinary tract abnormalities and anatomical differences related to the transplanted kidney are also risk factors for post-transplant UTIs [3,4]. Treatment with antibiotic therapy is crucial since untreated UTIs can result in sepsis, long-term damage to the allograft, or graft loss [2,5]. Recurrent UTIs, defined as ≥2 UTIs over six months or ≥3 UTIs over 12 months, are much more challenging to treat. Current literature suggests that anywhere from 2.9-27% of RTRs will develop recurrent UTIs [6]. Recurrent UTIs are of particular importance to the RTR population given their increased risk for allograft fibrosis and overall patient survival [7]. In one study, recurrent UTIs in RTR were responsible for 72% of total UTI-associated hospital admissions, and infection with multi-drug resistant bacteria was associated with the development of recurrent UTIs [6]. Of note, RTR is already at an increased risk for infection from drug-resistant organisms such as extended-spectrum beta-lactamase-producing *Enterobacterales* (ESBL), and it is estimated that one in 10 RTR will develop an ESBL UTI [7,8]. Recurrent UTIs are also associated with quality-of-life issues and effective treatment of recurrent UTIs improves quality of life, depression, and anxiety scores [9]. 

Management of recurrent UTIs involves addressing structural and functional abnormalities of the urinary tract along with antibiotic treatment [10]. Unfortunately, antibiotics have side effects, which include allergic reactions, antimicrobial resistance, and *Clostridium difficile* infection [11]. Treatment guidelines offer limited evidence for conservative management. Daily low-dose antibiotics for all patients and post-coital antibiotics for females have demonstrated efficacy in reducing the risk of recurrent UTIs. In post-menopausal women, vaginal estrogen is the most effective tool for preventing UTIs [12]. Nonetheless, these therapies are not 100% effective as there is still a risk of recurrence of UTIs despite adherence to therapy [13]. In RTR with recurrent UTIs, methenamine hippurate has been investigated as a treatment. While 47% of patients no longer had UTIs, 24% had a reduction in UTI frequency and 29% had no change [14]. Alternative solutions are needed for the management of recurrent UTIs, especially given the vulnerability of RTR to UTIs.

In our program, we use cystoscopy with bladder washout (BW) in the management of RTR with recurrent UTIs. The use of BW as a treatment for recurrent UTIs stemmed from incidental subjective reductions in the number of UTIs in RTR undergoing cystoscopy for any reason. Currently, literature on the use of BWs in the management of recurrent UTIs is sparse. Evidence has demonstrated that bladder instillation without antibiotics has a role in preventing UTIs in catheterized patients [15,16]. Another study showed that BWs with an anti-septic solution are effective for treating catheter-associated UTIs [17]. However, BW has yet to gain widespread adoption and is rarely used among urologists. More often, BWs have been used in research settings for localized UTIs of the genitourinary system [18-20]. Others have reported using BWs for radiation cystitis, genitourinary cancer, and even amphotericin B to treat canididuria [21,22]. In 2016, a retrospective study performed at our center investigating normal saline BW in RTR with a history of recurrent UTIs showed a 45.8% decrease in UTIs seen at six months following BW as well as a significant clearance rate of ESBL [23]. Since that time, all RTRs referred to the transplant urology service with recurrent UTIs have undergone cystoscopy and BW using normal saline as adjunctive treatment. This article was previously accepted for presentation as a meeting abstract at the 2023 annual American Urologic Association meeting on April 27th, 2023. We hypothesize that BW reduces the incidence and recurrence of UTIs in RTR. This study is a retrospective review of the effect of cystoscopy with normal saline BW on recurrent UTIs in RTR.

## Materials and methods

Study design

This was a retrospective study to evaluate the utility of BW procedures on renal transplant patients diagnosed with recurrent UTIs between December 2013 and July 2021 at a single institution. This study was approved by the Institutional Review Board (14051305-IRB01). Inclusion criteria were patients with a history of a renal transplant, and ≥2 UTIs in a six-month period prior to undergoing a BW. All UTIs that occurred six months before and after the BW procedure were treated with a 10-14-day course of culture-guided antibiotics. For patients who had multiple BWs in a one-year period, only the initial BW procedure was included as a data point. Patients with indwelling ureteral stents, foley catheters, or those that require intermittent catheterizations were excluded from the study. A UTI was defined as a urine culture growing >10^5 ^CFU/mL of bacteria with symptoms suggestive of an infection (e.g., dysuria, frequency, and urgency). All patients were started on a 10-14 day course of culture-guided oral or IV antibiotics peri-operatively, with the aim of performing the BW procedure in the mid-course of antibiotic therapy.

Per institutional practice, immunosuppression was reduced during the treatment of UTIs by holding antimetabolites; this applied to pre- and post-BW in all patients. Of note, there were no occurrences of acute graft rejection during the study period.

Data collection included demographics, renal transplant information, urinalyses, urine cultures (including organism and sensitivities), and antibiotic usage (including type, duration, setting, and route), in the six months preceding and following the BW procedure.

Primary and secondary outcomes

The primary outcome was the difference in the frequency of UTIs in the six months prior to a BW compared to the six months following a BW. A successful BW was defined as having zero to one UTI in the six months following the BW. In other words, patients in the pre-BW phase were the control group, while patients in the post-BW phase were the treatment group. Secondary outcomes included stratifying BW success based on infectious organisms, antimicrobial use, and length of time from renal transplant. We categorized organisms into three bacterial classes: *Enterobacterales, Enterococci,* and others. *Enterobacterales and Enterococci* were further divided based on antibiotic sensitivity (*Enterobacterales*: pan-sensitive, ESBL, or carbapenemase-producing; *Enterococci*: ampicillin-sensitive or VRE). Notably, no ampicillin-sensitive VRE was identified. 

Statistical analysis

Data was analyzed using t-tests, chi-square tests, and Fisher exact tests, as appropriate. Statistical significance was defined as p<0.05. Statistical analysis was performed using SAS v9.4 (SAS, Cary, NC). 

Bladder washout procedure

Preoperative antibiotics were chosen based on the patient's urine culture and sensitivities. Each subject underwent monitored anesthesia care (MAC). Subjects were then positioned in dorsal lithotomy position and then prepped with Betadine surgical scrub and draped in a sterile fashion. A rigid cystoscopy and urine collection were performed followed by a washout with cycles of filling and emptying the bladder with normal saline until 6L normal saline was used. Special attention is paid to cystoscope positioning to ensure complete filling and emptying. The bladder is emptied at the end of the procedure. All cases took less than 10 minutes to complete. All procedures were done on an outpatient basis.

## Results

A total of 106 patients were included in the study with a total of 118 BW performed. Patient demographic information is in Table 1. The BW success rate was significantly higher in patients identifying as white (80.65%) compared to patients who identified as Latino (51.28%). No other demographics had differences in BW success rates (Table 1). 

**Table 1:** Baseline characteristics and associated BW success BW, bladder washout

TABLE 1	Total (N=106)	BW success rate	p-value
Age (mean, range)	53.58 (23-81)		
Body mass index (mean, range)	27.76 (18-46)		
Gender (N, %)			0.365
Female	82 (77.36)	56 (69.41)	
Male	24 (22.64)	14 (60.00)	
Race (N, %)			0.033
White	30 (28.57)	24 (80.65)	
African American	28 (26.67)	21 (75.86)	
Latino	37 (35.24)	18 (51.28)	
Asian	10 (9.52)	7 (70.00)	
Diabetes (N, %)			0.994
Yes	61 (59.22)	37 (61.29)	
No	42 (40.78)	32 (77.78)	
Insulin use (N, %)			0.075
Yes	55 (57.29)	32 (58.18)	
No	43 (42.71)	31 (76.74)	
Kidney stones (N, %)			0.575
Yes	20 (19.80)	12 (60.00)	
No	81 (80.20)	54 (68.24)	
History of urinary reflux (N, %)			0.769
Yes	14 (13.73)	10 (71.43)	
No	88 (86.27)	58 (67.39)	

The mean number of UTIs was 2.76 (range, 1-7) before the BW and 1.16 (range, 0-5) after the BW. On average, there were 1.60 fewer UTIs in the post-BW period compared to the pre-BW period. The difference is statistically significant (Wilcoxon signed-rank test, p<0.0001) (Table 2).

**Table 2:** Overall BW success and change in the number of UTIs BW, bladder washout; UTIs, urinary tract infections

Table 2	Total (N=118)	p-value
BW (N, %)		N/A
Success	81 (68.64)	
Failure	37 (31.36)	
BW (N, range)		<0.0001
Pre-BW	2.76 (2-7)	
Post-BW	1.16 (0-5)	
Change in number of UTIs	-1.6 (-5-2)	

68.64% of patients were successfully treated with BW, meaning they no longer met the criteria for recurrent UTIs (<1 UTI) in the six-month post-BW period (Table 2). The treatment success rates by bacterial class based on immediate pre-BW UTI were 67%, 75%, and 71.43% for *Enterobacterales, Enterococci*, and other bacteria, respectively. There is no statistically significant difference in success rates stratified by bacterial class (p=1) (Table 3).

**Table 3:** Organisms identified and associated success with BW BW, bladder washout; UTIs, urinary tract infections

Table 3	Total	BW success rate	p-value
Bacterial class of UTI prior to washout (N, %), N=112			1.000
Enterobacterales	100 (86.96)	67 (67)	
Enterococcus	8 (6.96)	6 (75)	
Other	7 (6.09)	5 (71.43)	
Bacterial resistance class of UTI prior to washout (N, %), N=108			0.693
Pan-sensitive *Enterobacterales*	84 (77.78)	56 (66.67)	0.343
Extended-spectrum beta-lactamases	15 (13.89)	11 (73.33)	
Carbapenemase *Enterobacterales*	1 (0.93)	0 (0)	
Amp-sensitive *Enterococcus*	7 (6.48)	5 (71.43)	1.000
VRE	1 (0.93)	1 (100)	

The organisms causing UTIs were categorized into five antibiotic resistance classes: Pan-sensitive *Enterobacterales* (77.78%), ESBL *Enterobacterales* (13.89%), Carbapenemase-resistant *Enterobacterales* (0.93%), Ampicillin-sensitive *Enterococci* (6.48%), or VRE (0.93%). BW success rates for these classes were 66.67, 73.33%, 0%, 71.43%, and 100% respectively, demonstrating no statistically significant difference across bacterial resistance classes (p=0.6937) (Table 3). There was no difference among the three *Enterobacterales* resistance classes (p=0.3435) or between the two *Enterococci* resistance classes (p=1) (Table 3).

There was no statistically significant difference among different antibiotics used prior to BW (p=0.2656) or among different peri-operative antibiotics (p=0.5507) (Table 4). There were no post-operative complications relating to the BW procedure in any patient.

**Table 4:** Preoperative and peri-operative treatment for UTIs BW, bladder washout; UTIs, urinary tract infections

Table 4	Total (N=108)	BW success rate	p-value
Treatment for UTI immediately prior to BW (N, %)			0.265
Amoxacillin-Clavulonate	30 (27.78)	20 (66.67)	
Amoxicillin	1 (0.93)	0 (0)	
Cefdinir	1 (0.93)	1 (100)	
Cefepime	1 (0.93)	0 (0)	
Cefixime	2 (1.85)	1 (50)	
Ceftriaxone	2 (1.85)	1 (50)	
Cefuroxime	1 (0.93)	0 (0)	
Cephalexin	8 (7.41)	6 (75)	
Ciprofloxacin	1 (0.93)	1 (100)	
Doxycycline	1 (0.93)	0 (0)	
Ertapenem	7 (6.48)	4 (57.14)	
Fosfomycin	2 (1.85)	1 (50)	
Levofloxacin	38 (35.19)	30 (78.95)	
Meropenem	3 (2.78)	3 (100)	
Nitrofurantoin	5 (4.63)	5 (100)	
Trimethoprim-Sulfamethoxazole	5 (4.63)	4 (80)	
Peri-operative BW antibiotics (N, %)			0.5507
Amoxicillin/clavulanic acid	3 (2.56)	1 (33.33)	
Ampicillin	4 (3.42)	4 (100)	
Cefazolin	9 (7.69)	4 (44.44)	
Ceftriaxone	39 (33.33)	28 (71.79)	
Ciprofloxacin	1 (0.85)	1 (100)	
Clindamycin	1 (0.85)	1 (100)	
Daptomycin	1 (0.85)	1 (100)	
Doxycycline	1 (0.85)	0 (0)	
Ertapenem	10 (8.55)	6 (60)	
Levofloxacin	24 (20.51)	19 (79.17)	
Meropenem	4 (3.42)	3 (75)	
None	11 (9.40)	7 (63.64)	
Piperacillin-tazobactam	8 (6.84)	5 (62.50)	
Vancomycin	1 (0.85)	0 (0)	

## Discussion

To our knowledge, this study is the first to investigate the effectiveness of BW in the management of recurrent UTIs in RTR. The data showed that nearly 70% of RTR patients no longer met the criteria for recurrent UTIs after being treated with BW and that the mean number of UTIs/patients decreased by 1.6 in the six months following BW. This data highlights the utility of BW in RTR patients who suffer from recurrent UTIs, despite appropriate antibiotic treatment.

The most common pathogenic bacteria causing UTIs in this cohort was *Escherichia coli*, but many infections were caused by antibiotic-resistant bacteria (i.e., ESBL and* *VRE). The success rates between patients with antibiotic-sensitive versus resistant bacteria did not significantly differ. Additionally, no differences were found in the success rates of the BW based on the type of antibiotic used peri-operatively. Our study was likely underpowered to detect differences if they did exist.

The mechanism by which a BW can reduce the incidence of UTIs in RTR is not well studied. RTRs are at an increased risk of recurrent UTIs due to complicated anatomy and multiple comorbidities. Compounding these risks is the prevalence of voiding dysfunction following a renal transplant. One study found that 19-27% of male RTRs experienced voiding dysfunction post-operatively, such as bladder outlet obstruction, bladder neck contracture, urethral stricture, and detrusor underactivity [24]. Furthermore, it is known that high residual volumes are associated with an increased risk of UTIs [25]. RTRs who have a weakened host defense from immunosuppression medications are likely unable to completely eradicate pathogenic bacteria in the bladder contributing to recurrent infections. By performing a BW, we are able to improve source control and reduce the number of bacteria for antibiotics to act on.

The possibility of a novel treatment for recurrent UTIs is significant. An RCT comparing daily low-dose antibiotic prophylaxis vs placebo and the effect of UTI recurrence over 12 months demonstrated a 48% reduction in UTI frequency in the prophylaxis group [26]. The treatment group suffered 1.3 UTIs per person-year, which was a significant reduction from the control group. However, patients were still suffering symptomatic UTIs despite prophylaxis. Antibiotic prophylaxis was also associated with a significantly increased risk for anti-microbial resistance compared to the control group. Side effects attributed to antibiotic prophylaxis included GI upset, rash, and Candida infection [26]. Other research has shown that cessation of daily low-dose prophylaxis or post-coital antibiotics returns patients to pre-treatment UTI rates [27]. The use of BW, which does not depend on long-term antimicrobial therapy, lessens the risk of bacterial resistance and eliminates the potential side effects of frequent antibiotic administration. Risks of BW are mainly limited to anesthesia; however, some patients need to undergo anesthesia for routine cystoscopy in the workup of recurrent UTIs.

Recently, a sublingual vaccine was introduced as a treatment option for women with recurrent UTIs. This novel vaccine was shown to significantly increase UTI-free rates over a six-month period compared to antibiotic-prophylaxis alone (35%-90% and 0%, respectively) [28]. Although not yet studied on RTR, this promising new treatment option highlights the desire to improve the current treatment paradigm for recurrent UTIs.

Although this study is focused on RTR, other populations of patients with recurrent UTIs and voiding dysfunction may benefit from BWs. Pathologies ranging from neurogenic bladder to benign prostatic hyperplasia can be complicated by high residual volumes and recurrent UTIs [29]. We suspect that these patients may benefit from BW in addition to antibiotic treatment for the management of recurrent UTIs. In our analysis, we identified two patients who had lower urinary tract stones at the time of cystoscopy and BW. Both patients no longer met the criteria for recurrent UTIs following BW highlighting the role that stones play as nidus of infection. 

Despite the promising success rate of BWs for recurrent UTIs, 30% of patients had more than one UTI in the six-month follow-up period. Multiple mechanisms contribute to the pathophysiology of recurrent UTIs. Uropathogens can ascend into the bladder and can attach to the uroepithelial surface via P Fimbrae and fimH. Intracellular bacteria can then replicate within epithelial cells, evading urine flow and the immune response [30]. We suspect that bacteria that are able to anchor themselves to the uroepithelium and replicate within the cells of the bladder wall were resistant to the BW procedure and led to recurrent UTIs in those patients. Other potential reasons include uncorrected structural or functional abnormalities such as neurogenic bladder and accompanying high post-void residuals, vesicoureteral reflux, and retained foci of infection [10].

Our study was limited by its retrospective nature and a relatively short follow-up period of six months. We use a six-month period to diagnose recurrent UTIs in our clinical practice, which led us to choose the six-month pre-BW period for this study. We decided on the six-month post-BW follow-up period to have consistency between the pre- and post-BW periods. While we suspect that the benefits of the BW were maintained over a longer period, this needs to be investigated in future studies. Another subjective finding was the not infrequent change in bacteria and/or antibiotic sensitivity patterns post-BW that may be easier to treat; this also warrants further investigation. It is noteworthy that while many cystoscopies can be performed in a clinical setting, the BW procedures were done in the operating room with an anesthesiologist administering MAC so that a rigid cystoscope could be used, which allowed complete emptying of the bladder in-between fillings; this was considered to be crucial to the success of the procedure. Secondary endpoints such as difference in BW success based on infection with MDR organisms did not reach significance. The small sample sizes may explain the lack of difference in success based on bacterial resistance. Our results warrant further prospective studies examining the short-, mid-, and long-term effects of BWs in RTR, in addition to other populations suffering from recurrent UTIs.

## Conclusions

In our study population, BWs decreased the incidence of UTIs in the six-month post-operative period as nearly 70% of patients did not have UTI recurrence. This data provides evidence that BW may have utility in transplant recipients with recurrent UTIs. We hope this will stimulate further prospects.
